# First Detection of SARS-CoV-2 B.1.617.2 (Delta) Variant of Concern in a Symptomatic Cat in Spain

**DOI:** 10.3389/fvets.2022.841430

**Published:** 2022-04-01

**Authors:** Sandra Barroso-Arévalo, Lidia Sánchez-Morales, Marta Pérez-Sancho, Lucas Domínguez, José M. Sánchez-Vizcaíno

**Affiliations:** ^1^VISAVET Health Surveillance Center, Complutense University of Madrid, Madrid, Spain; ^2^Department of Animal Health, Faculty of Veterinary, Complutense University of Madrid, Madrid, Spain

**Keywords:** SARS-CoV-2, cats, pet, delta variant, transmission

## Abstract

Natural and experimental SARS-CoV-2 infection in pets has been widely evidenced since the beginning of the COVID-19 pandemic. Among the numerous affected animals, cats are one of the most susceptible species. However, little is known about viral pathogenicity and transmissibility in the case of variants of concern (VOCs) in animal hosts, such as the B.1.617.2 (Delta) variant first detected in India. Here, we have identified the B.1.617.2 (Delta) VOC in a cat living with a COVID-19 positive owner. The animal presented mild symptoms (sneezing) and a high viral load was detected in the oropharyngeal swab, suggesting that an active infection was occurring in the upper respiratory tract of the cat. Transmission from the owner to the cat occurred despite the human being fully vaccinated against SARS-CoV-2. This study documents the first detection of B.1.165.2 VOC in a cat in Spain and emphasizes the importance of performing active surveillance and genomic investigation on infected animals.

## Introduction

Since December 2019, a new virus denominated severe acute respiratory syndrome coronavirus 2 (SARS-CoV-2), has threatened the entire world. This virus, the causative agent of the disease named COVID-19, is an enveloped single-stranded RNA virus belonging to the *Coronaviridae* family, *Beta* genus (1). The viral genome includes 13 open reading frames (ORFs) and four major structural proteins: the surface Spike (S) protein, the envelope (E) protein, the matrix protein (M), and the nucleocapsid (N) protein (2). Following the global spread of SARS-CoV-2, the emergence of new variants of the virus has kept the world on tenterhooks. Among the numerous variants, some of them have been proved to be even more hazardous than the original strain. These variants, denominated as variants of concern (VOCs), present higher transmission rates and a more effective evasion of the host immune system, which makes it more difficult the adequated control of the disease. The first determined VOC was the B.1.1.7 (20I/N501Y.V1), which was identified in England (3). After this event, a second variant with the N501Y mutation was first detected in South Africa, named B.1.351 (20J/N501Y.V2) (4), and lately, the P.1 variant was reported in Brazil (20I/N501Y.V3) (5). Despite their hazardous properties, vaccination implementation helped to control the damages caused by these variants. However, since late March 2021, India started experiencing an increase in the number of COVID-19 cases reaching more than 400,000 cases and 4,000 deaths reported each day in early May 2021 (6). These fatalities were associated with a new lineage, the B.1.617.2 (Delta) variant, that was first detected in India in December 2020 and became the most commonly reported variant in the country by mid-April 2021. The delta variant is characterized by the spike protein mutations T19R, Δ157-158, L452R, T478K, D614G, P681R, and D950N. Several of these mutations may alter host immune response and increase viral replication, leading to higher viral loads and increased transmission rates (7). Currently, the Delta variant has been replaced by a new heavily mutated variant known as B.1.1.529 (BA) (Omicron) (8), first reported in South Africa on November 24, 2021 (9). This new variant has dominated the current epidemiologic scenario, replacing the present Delta prevalence around 4.2% in Spain (10).

Although the concern regarding the delta variant in the human population is widely documented, little is known about the transmission capacity and the effects of this variant in animals. Due to the zoonotic origin of the COVID-19 disease, numerous experimental and field studies have been conducted in order to explore the extent of the infection in animals and to elucidate their role as reservoirs (11–15). Concretely, efficient SARS-CoV-2 transmission between owners and their pets has been demonstrated worldwide (16–18). Among the different animals affected, cats have been shown to be one of the more susceptible species (19). One proof of this is the high number of SARS-CoV-2 sequences retrieved from cats submitted to the GISAID website, which corresponds with 119 sequences from different parts of the world. In addition, several studies have reported natural infection with SARS-CoV-2 in this species (15, 17, 18). We cannot dismiss, therefore, the potential role of cats as intermediate hosts of the virus, which may trigger the development of new mutations in the viral genome. Moreover, genomic surveillance in infected pets has evidenced animal infection with at least one VOC, the B.1.1.7 alpha variant (20–22). These events highlight the importance of performing genetic investigations on samples from infected cats in order to understand the transmission and evolution of the virus in these hosts.

To the best of our knowledge, here, we documented the first human to domestic cat transmission of the SARS-CoV-2 B.1.617.2 (delta) variant in Spain. Although the cat only presented subtle clinical signs, it showed high levels of viral RNA in the oropharyngeal swab taken. These facts suggest an active viral infection and open the question of whether the cat would act as a source of virus.

## Materials and Methods

### Animal Sampling and Clinical Inspection

Cat sampling was conducted on 3th October during the owner quarantine period, 9 days after COVID-19 positive confirmation of the owner. Oropharyngeal and rectal swabs, as well as feces, were collected in DeltaSwab® Virus 3 ml contained in viral transport media (VTM) (Deltalab S.L., Cataluña, Spain) using protocols approved by the Complutense University of Madrid's Ethics Committee for Animal Experiments (Project License 14/2020). Serum sample was collected in a tube without any anti-coagulant 65 days after the initial sampling in order to evaluate the presence of neutralizing antibodies.

During animal sampling, the cat was evaluated by the veterinarian looking for clinical signs compatible with SARS-CoV-2 infection, such as apathy, nasal discharge, cough, or sneezing. In addition, the owner was surveyed in order to identify potential clinical signs.

### Detection of SARS-CoV-2 Infection by Reverse Transcription-Quantitative PCR and Virus Isolation

RNA from these swabs was extracted using the KingFisher Flex System automated extraction instrument (ThermoFisher, Waltham, MA, USA), with the MagMAX Viral/Pathogen Nucleic Acid Isolation Kit (ThermoFisher), according to the manufacturer's instructions. The detection of SARS-CoV-2 RNA was performed using the envelope protein (E)-encoding gene (Sarbeco) and two targets (IP2 and IP4) of the RNA-dependent RNA polymerase gene (RdRp) in an RT-qPCR protocol established by the World Health Organization according to the guidelines that can be found at https://www.who.int/emergencies/diseases/novel-coronavirus-2019/technical-guidance/laboratory-guidance (23).

Viral isolation was performed using the previously described methods in Gortázar et al. (24).

### Virus Neutralization Test

Serum was tested for neutralizing antibodies against SARS-CoV-2 by means of a VNT, according to the methods previously described in Barroso-Arévalo et al. (15).

### Whole-Genome Sequencing and Phylogenetic Analysis

Whole-genome sequence was obtained from the positive oropharyngeal sample by RT-PCR using 38 primers sets according to the protocol described by Paden et al. (25). The 38 purified amplicons obtained were finally sequenced with 2x coverage using the Sanger dideoxy method (Applied Biosystems). Each primer's corresponding sequence was trimmed. Raw sequence data were aligned and edited using the Sequencing Analysis software v.5.3.1 (Applied Biosystems). Sequence assembly was performed using the SeqScape v.2.5 software (Applied Biosystems), employing the SARS-CoV-2 isolate Wuhan-Hu-1, complete genome (GenBank accession number: NC_045512) as a reference genome.

MEGA X software (26) was used for the phylogenetic analysis. The analysis included a total of 35 representative sequences, including sequences from cats and dogs, the reference genome from Wuhan, as well as variants of concern (B.1.1.7, P.1, B.1.351, and B.1.617.2). The final alignment involved 36 whole-genome sequences with an average amino acid p-distance (1-amino acid identity) of 0.011, which is considered adequate since it is within the acceptance threshold of <0.8 (26). This alignment was used to build the phylogenetic tree using the maximum likelihood method, the Subtree-Pruning-Regrafting (SPR) algorithm, and bootstrap testing of 2,000 replicates. Since only those bootstrap values ≥ 70% are considered valid, a consensus tree was computed, accepting the default 50% cut-off value, according to Hall, BG (27), in such a way that several clades are shown as a polytomy.

The presence of mutations was evaluated using the CoVsurver mutations app available on the GISAID website (https://www.gisaid.org/) (accessed on 20, October 2021). We appreciatively acknowledge the different laboratories and funders of GISAID for offering these SARS-CoV-2 sequences.

## Results

### Clinical Case Description

The cat, a common European 7-year-old cat, was living with a confirmed COVID-19 positive owner during her whole quarantine period. The owner was fully vaccinated when became infected and presented mild symptoms of the disease. The only clinical sign of the cat reported by the owner was sneezing. The cat did not show any other symptoms during the veterinarian inspection.

### RT-qPCR and Viral Isolation Results

RT-qPCR and viral isolation results are shown in Table 1.

**Table 1:** SARS-CoV-2 test results for a pet cat from Madrid (Spain) that was confirmed for infection with the B.1.617.2 variant of concern (VOC).

**Animal ID, date of sample collection**	**RT-qPCR Ct values for swab testing**				**Viral isolation**
	**Sample type**	**RT-qPCR target**			
		**Sarbeco**	**IP2**	**IP4**	
Cat_2162, October 3th, 2021	Oropharyngeal swab	24.09	24.51	26.24	Negative
	Rectal swab	38.6	ND	ND	NA
	Feces	ND	39.13	ND	NA

### Neutralizing Antibodies Detection by Employing VNT

Serum sample taken 65 days after the initial sampling showed neutralizing antibodies using VNT, with a titer of 1/512, which corresponds with a high amount of neutralizing antibodies and is correlated with clinical protection from SARS-CoV-2 infection (28).

### Whole-Genome Sequencing and Phylogenetic Analysis

**Figure 1:**
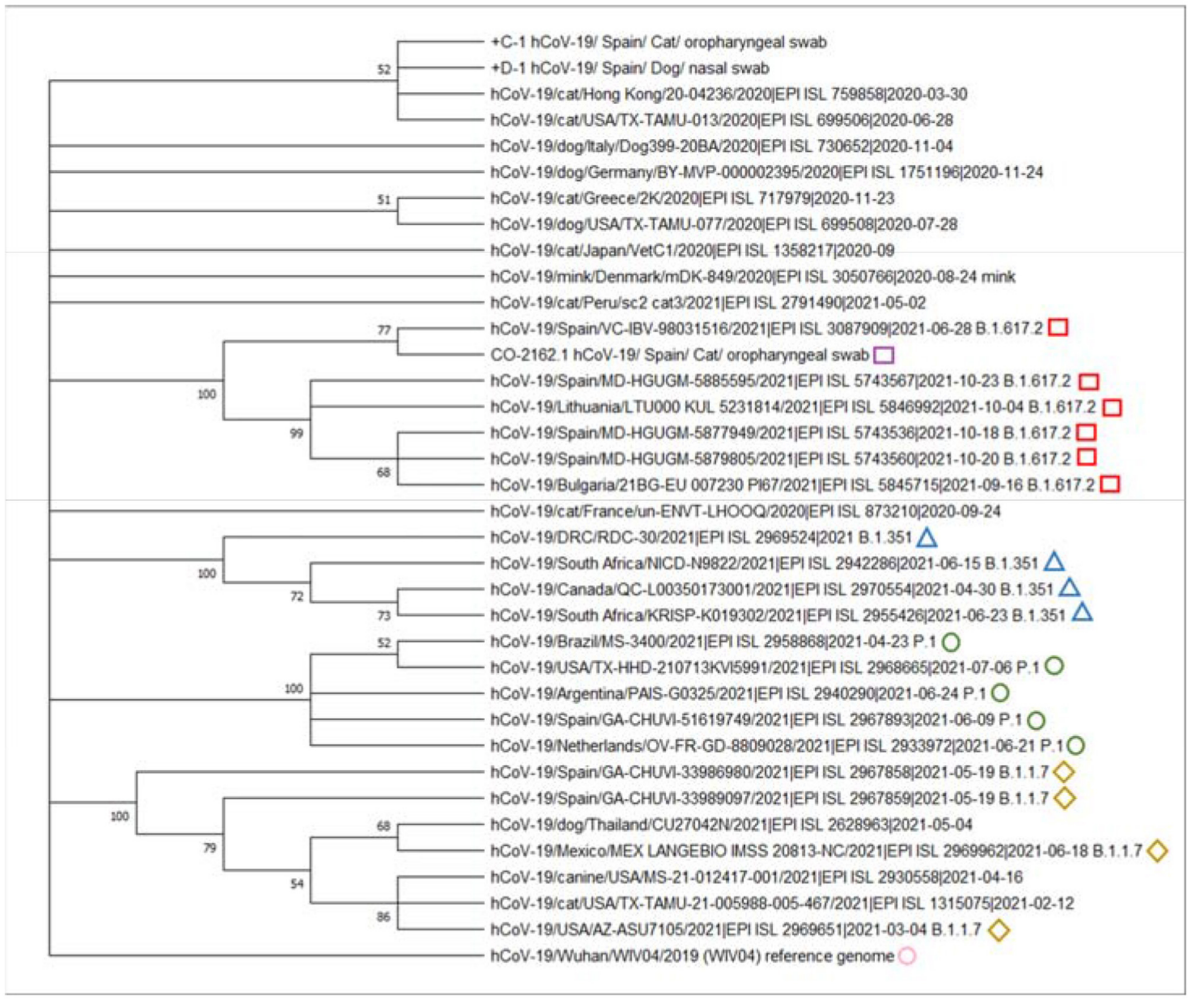
Phylogenetic analysis of SARS-CoV-2 (Severe Acute Respiratory Syndrome Coronavirus 2) indicated that the whole-genome sequence from this study (purple square) was similar and clustered with the SARS-CoV-2 B.1.617.2 (Delta) sublineage genomes from included in the alignment. Green squares indicate the variant of concern B.1.1.7; red diamonds indicate the variant of concern P.1; blue triangles indicate the variant of concern B.1.617.2; yellow circles indicate the variant of concern B.1351. Pink circle indicates the reference SARS-CoV-2 (WIV04) genome from Wuhan. We appreciatively acknowledge the different laboratories and funders of GISAID for offering these SARS-CoV-2 sequences (Supplementary Material 1).

The maximum likelihood based on the general time-reversible model (26) was used for inferring the evolutionary relationships among the different whole-genome sequences. No sequence was available from the owner, so it could not be included into the alignment. A total of 32 nucleotide sequences, including 1st, 2nd, 3rd, and noncoding codon positions were analyzed. In order to avoid the inclusion of alignment gaps, missing data, and ambiguous bases, positions with <95% site coverage were removed from the alignment, resulting in the analysis of 29,514 positions. The evolutionary history of the analyzed sequences was obtained from the bootstrap consensus tree deducted from 2,000 replicates (29). First, initial tree(s) were obtained automatically using the neighbor-joining and BioNJ algorithms to a matrix of pairwise distances estimated using maximum composite likelihood and then choosing the topology with a better log-likelihood value. For modelization of differences in the rate of evolution among different sites, a discrete gamma distribution (two categories, +G parameter = 0.059) was used. The resulting phylogenetic tree is shown in Figure 1. As observed in the phylogenetic tree, the genome sequence from this study (CO-2162.1) clustered with sequences belonging to the B.1.617.2 (Delta) sublineage.

Analysis in the CoVsurver mutations app (GISAID) revealed that the sequence presented 30 mutations (Table 2).

**Table 2:** List of mutations displayed in the different regions of the genome of SARS-CoV-2 in the sequence obtained in this study.

**Location in the genome**	**Mutations displayed**
NSP3 (ORF1a)	P822L
NSP4 (ORF1a)	A446V
NSP6 (ORF1a)	A41V, V149A, T181I
NSP12 (ORF1b)	R197Q, P323L, G671S
NSP13 (ORF1b)	P77L
NSP14 (ORF1b)	T16I
Spike	T19R, G142D, E156G, F157del, R158del, A222V, T250I, S255F, L452R, T478K, D614G, P681R, D950N
NS3	S26L
M	I82T
NS7a	V82A, T120I
N	D63G, R203M, D377Y

*NSP, Non-structural protein; NS3, Non-structural protein 3; M, Membrane protein; NS7a, Accessory protein 7a; N, Nucleocapside protein*.

## Discussion

This is, to our knowledge, the first report of the B.1.617.2 (Delta) VOC in a cat pet worldwide, which confirms the transmission of this variant can occur between infected people and their pets, at least in the case of cats. This variant, which is currently the most prevalent SARS-CoV-2 strain has shown higher transmission rates even in vaccinated people causing a decrease in vaccine effectiveness (30, 31). For these reasons, recent vaccine effectiveness studies now focus on this variant (32, 33). All these facts evidence the importance of exploring the reach of this variant in all the possible scenarios, and, therefore, to know if this VOC can infect pets which may act as a potential source of infection. In addition, the emergence of new variants associated with animal infections cannot be dismissed, as has previously occurred in the case of minks (34, 35).

In this study, we investigated a reverse zoonosis event in a pet cat living with a COVID-19 positive owner. The cat presented light symptoms of the disease (sneezing) and was sampled during the quarantine period of its owner. Molecular analysis was performed in several samples from the cat and SARS-CoV-2 infection was confirmed by RT-qPCR, following a genomic investigation from the positive sample (oropharyngeal swab). Thus, the phylogenetic assay revealed that the sequence from the cat presented the mutations proper of the B.1.617.2 (delta) VOC. It is also noteworthy that transmission between the infected human and the cat occurred despite the owner having a complete vaccination schedule. This finding alerts us about the high transmission capacity of this VOC, which avoided all the boundaries and jumped from the owner to the pet. The animal also developed neutralizing antibodies, which demonstrates that an active infection occurred following an effective immune system response.

Although numerous human-based studies have demonstrated the dramatic consequences associated with infection by this VOC in humans (36, 37), little is known about its impact on animal infection. Several field studies have previously demonstrated the presence of infection with the B.1.1.7 VOC in pets (20–22), observing associated symptoms such as cardiomyopathies in some of these cases (21).

The delta variant has been reported to be even more transmissible than other VOCs previously detected, such as the B.1.1.7 variant (33, 38). In addition, vaccine effectiveness seems to be decreased in the case of infection with this variant, as some reports have evidence (32, 38). This fact may explain that the owner of the infected cat reported in this study was able to transmit the virus to the pet, despite being completely vaccinated. Although the virus was not isolated from the cat sample, a high viral load was detected in the animal's oropharyngeal swab, that allude to an active infection at the time of sampling. In addition, the cat had compatible symptoms with the disease, without any known comorbidities. Taking into account that experimental studies have reflected that cats often do not present clinical signs when become infected (39), the presence of sneezing in this cat, may suggest a higher virulence of the isolate. Under this scenario, the role of the cat as an active source of infection cannot be dismissed, as well as the potential capacity of the virus to mutate into the animal. All these facts together highlight the risk associated with pet delta variant infection and underline the importance of performing active surveillance in pets living with COVID-19 infected people, including genomic investigation in order to detect infections with VOCs or potential mutations associated with animal hosts.

## Data Availability Statement

The datasets presented in this study can be found in online repositories. The names of the repository/repositories and accession number(s) can be found at: https://www.ncbi.nlm.nih.gov/genbank/, OL336792.

## Ethics Statement

The animal study was reviewed and approved by Complutense University of Madrid's Ethics Committee for Animal Experiments (Project License 14/2020). Written informed consent was obtained from the owners for the participation of their animals in this study.

## Author Contributions

SB-A and LS-M performed the sampling, veterinary inspection, and laboratory analysis and wrote the initial manuscript. LD and JS-V acquired the funds. MP-S, LD, and JS-V reviewed the manuscript. All authors have read and approved the final version of the manuscript.

## Funding

The Institute of Health Carlos III (ISCIII) was the project Estudio del potencial impacto del COVID19 en mascotas y linces founder (reference: COV20/01385).

## Conflict of Interest

The authors declare that the research was conducted in the absence of any commercial or financial relationships that could be construed as a potential conflict of interest.

## Publisher's Note

All claims expressed in this article are solely those of the authors and do not necessarily represent those of their affiliated organizations, or those of the publisher, the editors and the reviewers. Any product that may be evaluated in this article, or claim that may be made by its manufacturer, is not guaranteed or endorsed by the publisher.
